# Psychological understanding of Anorexia nervosa gained from combined clinical care

**DOI:** 10.1192/j.eurpsy.2024.1155

**Published:** 2024-08-27

**Authors:** H. Szilárd, X. Gonda, Á. Menus, J. Bognár, D. Pólya, J. Biliczki, Z. Bana, Z. Nemoda, J. Réthelyi

**Affiliations:** ^1^Psychiatry and Psychotherapy; ^2^Pediatrics Clinic no.1.; ^3^Faculty of Medicine, Semmelweis University; ^4^Psychiatry, Peterfy Hospital; ^5^Clinical Psychology; ^6^Molecular Biology, Semmelweis University, Budapest, Hungary

## Abstract

**Introduction:**

Anorexia nervosa (AN) is a debilitating illness with rapidly increasing incidence. The longer it lasts the more difficult is to cure. Although in the majority of the cases the main treatment is psychotherapy severe cases require inpatient admission for life saving support. The presentation expounds the combined therapeutic approach that anorexia nervosa patients of Semmelweis University Psychiatry and Psychotherapy Department provided with.

Currently the backbone of psychotherapy *for AN* is cognitive behavioral therapy (CBT), schema therapy and katathym imaginative psychotherapy (KIP) combined with psychodrama.

**Objectives:**

One of the aims of our research is to identify the most relevant focus of psychotherapy by identification of specific personality traits in patients with AN, who will be compared with healthy controls. Furthermore, two subgroups of patients will be compared with each other: the milder version of AN (BMI above 16) with the more severe form of AN who are required inpatient admission.

**Methods:**

Women with AN (age:18-45) have been compared to age-matched controls on MINI and SCID-5-AMPD interview variables and on the scales of online questionnaires, such as EDI-I., MZQ, DIS-Q, SCL-90, PHQ-9, STAI, CTQ and YPI.

**Results:**

Clinical care highlighted important underlying psychological causes such as inadequate mirroring, absence of the father, or on the contrary, overly intimate relationship with the father, and relentless inner voice as a consequence of unintegrated inner aspects.

The SCID-5-AMPD pointed out affected areas of personality such as Identity, Self-directedness, Negative affectivity, Intimacy, Alienation. Importantly, neither trauma scales (measured by CTQ), nor dissociation (measured by DIS-Q) differed significantly between patients and healthy controls.

**Conclusions:**

Planning psychotherapy could benefit from the identified foci. Anti-depressive medication must be considered in order to improve outcome of inpatient admission. The CTQ probably does not measure the subtle but chronic inadequacy of attachment and mirroring that apparently are typical in AN. The reliable identification of the typical dissociative inner voice that often seen in anorexia nervosa may need another questionnaire apart from DIS-Q.

***The research is supported by bilateral science and technology (S&T) cooperation project 2019-2.1.11-TÉT-2020-00242***

**Disclosure of Interest:**

None Declared

